# Deltaline from *Delphinium delavayi* Franch

**DOI:** 10.1107/S1600536811001681

**Published:** 2011-01-22

**Authors:** Xiong-Qing Wang, Qin Song, Xiao-Qiang Guo, Jun Yan

**Affiliations:** a Mianyang Normal University, Mianyang 621000, People’s Republic of China; b Institute of Biological Industry of Chengdu University, Chengdu 610016, People’s Republic of China

## Abstract

The title compound [systematic name: 6β,10-dihy­droxy-1α,14α,16β-trimeth­oxy-4-methyl-7β,8-(methyl­enedi­oxy)-20-ethyl­aconitan-6-yl acetate], C_27_H_41_NO_8_, is a C_19_-diterpenoid alkaloid and a major diterpenoid alkaloid component of the roots of *Delphinium delavayi* Franch. var. pogonanthum (Hand.-Mazz.) W. T. Wang. The mol­ecule has a lycoctonine carbon-atom skeleton with four six-membered rings and three five-membered rings among; three of the six-membered rings adopt chair conformations with the fourth adopting a boat conformation while all of the five-membered rings exhibit envelope conformations. Inter­molecular O—H⋯O hydrogen bonding is present in the crystal structure.

## Related literature

For the isolation of the compound from plants of the genus *Delphinium delavayi* Franch, see: Pelletier *et al.* (1980 ▶). For a related compound, see: Wang *et al.* (2009 ▶).
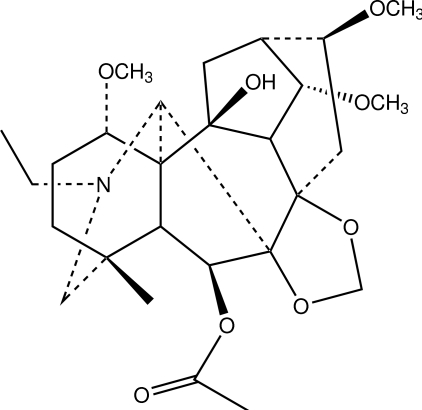


## Experimental

### 

#### Crystal data

C_27_H_41_NO_8_
*M* *_r_* = 507.61
Orthorhombic, 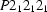

*a* = 8.5708 (3) Å
*b* = 16.3149 (5) Å
*c* = 18.5346 (6) Å
*V* = 2591.73 (16) Å^3^
*Z* = 4
Mo *K*α radiation
μ = 0.10 mm^−1^
*T* = 293 K
0.54 × 0.52 × 0.50 mm

#### Data collection

Xcalibur, Eos diffractometer
8392 measured reflections
3005 independent reflections
2270 reflections with *I* > 2σ(*I*)
*R*_int_ = 0.019

#### Refinement

*R*[*F* ^2^ > 2σ(*F* ^2^)] = 0.038
*wR*(*F* ^2^) = 0.085
*S* = 1.11
3005 reflections
332 parameters
H-atom parameters constrained
Δρ_max_ = 0.27 e Å^−3^
Δρ_min_ = −0.20 e Å^−3^

### 

Data collection: *CrysAlis PRO CCD* (Oxford Diffraction, 2009 ▶); cell refinement: *CrysAlis PRO CCD*; data reduction: *CrysAlis PRO RED* (Oxford Diffraction, 2009 ▶); program(s) used to solve structure: *SHELXTL* (Sheldrick, 2008 ▶); program(s) used to refine structure: *SHELXTL*; molecular graphics: *OLEX2* (Dolomanov *et al.*, 2009 ▶); software used to prepare material for publication: *OLEX2*.

## Supplementary Material

Crystal structure: contains datablocks I, global. DOI: 10.1107/S1600536811001681/xu5116sup1.cif

Structure factors: contains datablocks I. DOI: 10.1107/S1600536811001681/xu5116Isup2.hkl

Additional supplementary materials:  crystallographic information; 3D view; checkCIF report

## Figures and Tables

**Table 1:** Hydrogen-bond geometry (Å, °)

*D*—H⋯*A*	*D*—H	H⋯*A*	*D*⋯*A*	*D*—H⋯*A*
O2—H2⋯O7^i^	0.82	2.58	3.311 (2)	150

Symmetry code: (i) 
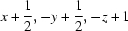
.

**Fig. 1.**
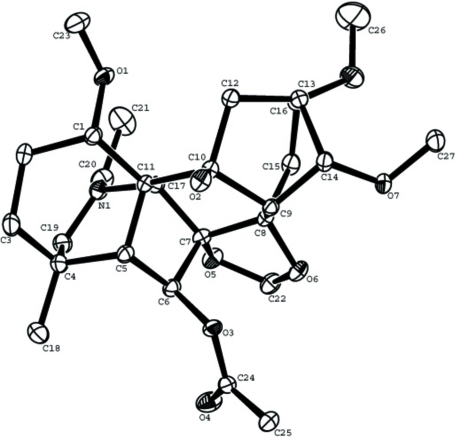
The molecular structure of the title compound with 30% probabiliy displacement ellipsoids for no-H atoms.

## supplementary crystallographic information

### Comment

The title compound, deltaline, is a diterpenoid alkaloid and had been previously isolated from plants of the genus *Delphinium delavayi* Franch (Pelletier,*et al.*, 1980), and its structure was established from the NMR and MS data. The compound itself has analgesic properties, and plants of the genus *Delphinium delavayi* Franch have also been therapeutically used to treat rheumaticpain, paralysis due to stroke, rheumatoid arthritis. In order to obtain further evidence for the exact configuration and conformation of the title compound, we have here determined its crystal structure. The naming and the rings conforming referred to the literature (Wang *et al.*, 2009).

The molecular structure of the title compound is shown in Fig. 1. Six-membered rings A (C1/C2/C3/C4/C5/C11) and B (C7/C8/C9/C10/C11/C17) adopt chair conformations; six-membered ring D (C8/C9/C14/C13/C16/C15) adopt an envelope conformation; six-membered N-containing heterocyclic ring E (C4/C5/C11/C17/N1/C19) displays the same chair conformation; five-membered rings C (C9/C10/C12/C13/C14) and F (C5/C6/C7/C17/C11) adopt an envelope conformation. While the five-membered N-containing heterocyclic G (O5/C7/C8/O6/C22) displays an envelope conformation.

The crystal structure contains intramolecular O—H···O hydrogen bonds between the hydroxy group and thecarbonyl O atom (Table 1).

The compound has the similar molecular skeleton with lycoctonine, they all belong to lycoctonine-type C_19_-diterpenoid alkaloid. The differences between them are different substituents.

### Experimental

Air-dried and powdered roots of *Delphinium delavayi* Franch (1000 g) were percolated with 0.1 *M* HCl (10 l) for 8 h. The obtained acid aqueous solution was basified with 10% aqueous NH_4_OH to pH 10 and then extracted with ethyl acetate (10 l×3). Removal of the solvent under reduced pressure afforded the total crude alkaloids (4.8 g) as a yellowish amorphous powder, which was chromatographed over a silica gel column, eluting with cyclohexane-acetone (9:1→1:2) gradient system, to afford deltaline (208 mg). The crystals suitable for X-ray structure analysis were obtained by slow evaporation from an acetone solution at room temperature.

### Refinement

H atoms were located geometrically with O—H = 0.82 and C—H = 0.96–0.98 Å, and refined using a riding model with *U*iso(H) = 1.2*U*eq(C) and 1.5U_eq_(O). The absolute configuration was not determined owing to the absence of strong anomalous scatterings; Friedel pairs were merged.

### Crystal data

**Table:**

C_27_H_41_NO_8_	*F*(000) = 1096
*M**_r_* = 507.61	*D*_x_ = 1.301 Mg m^−^^3^
Orthorhombic, *P*2_1_2_1_2_1_	Mo *K*α radiation, λ = 0.7107 Å
Hall symbol: P 2ac 2ab	Cell parameters from 4328 reflections
*a* = 8.5708 (3) Å	θ = 3.2–29.1°
*b* = 16.3149 (5) Å	µ = 0.10 mm^−^^1^
*c* = 18.5346 (6) Å	*T* = 293 K
*V* = 2591.73 (16) Å^3^	Block, colourless
*Z* = 4	0.54 × 0.52 × 0.50 mm

### Data collection

**Table:**

Xcalibur, Eos diffractometer	2270 reflections with *I* > 2σ(*I*)
Radiation source: fine-focus sealed tube	*R*_int_ = 0.019
graphite	θ_max_ = 26.4°, θ_min_ = 3.2°
Detector resolution: 16.0874 pixels mm^-1^	*h* = −10→6
ω scans	*k* = −20→20
8392 measured reflections	*l* = −23→21
3005 independent reflections	

### Refinement

**Table:**

Refinement on *F*^2^	Primary atom site location: structure-invariant direct methods
Least-squares matrix: full	Secondary atom site location: difference Fourier map
*R*[*F*^2^ > 2σ(*F*^2^)] = 0.038	Hydrogen site location: inferred from neighbouring sites
*wR*(*F*^2^) = 0.085	H-atom parameters constrained
*S* = 1.11	*w* = 1/[σ^2^(*F*_o_^2^) + (0.0438*P*)^2^] where *P* = (*F*_o_^2^ + 2*F*_c_^2^)/3
3005 reflections	(Δ/σ)_max_ < 0.001
332 parameters	Δρ_max_ = 0.27 e Å^−^^3^
0 restraints	Δρ_min_ = −0.20 e Å^−^^3^

### Special details

**Table:**

Geometry. All e.s.d.'s (except the e.s.d. in the dihedral angle between two l.s. planes) are estimated using the full covariance matrix. The cell e.s.d.'s are taken into account individually in the estimation of e.s.d.'s in distances, angles and torsion angles; correlations between e.s.d.'s in cell parameters are only used when they are defined by crystal symmetry. An approximate (isotropic) treatment of cell e.s.d.'s is used for estimating e.s.d.'s involving l.s. planes.
Refinement. Refinement of *F*^2^ against ALL reflections. The weighted *R*-factor *wR* and goodness of fit *S* are based on *F*^2^, conventional *R*-factors *R* are based on *F*, with *F* set to zero for negative *F*^2^. The threshold expression of *F*^2^ > σ(*F*^2^) is used only for calculating *R*-factors(gt) *etc*. and is not relevant to the choice of reflections for refinement. *R*-factors based on *F*^2^ are statistically about twice as large as those based on *F*, and *R*- factors based on ALL data will be even larger.

### Fractional atomic coordinates and isotropic or equivalent isotropic displacement parameters (Å^2^)

**Table:**

	*x*	*y*	*z*	*U*_iso_*/*U*_eq_	
O1	0.7652 (2)	0.55926 (11)	0.35593 (8)	0.0456 (5)	
O2	0.81403 (19)	0.35689 (10)	0.45254 (9)	0.0390 (4)	
H2	0.7998	0.3319	0.4904	0.059*	
O3	0.5153 (2)	0.37548 (9)	0.59848 (8)	0.0380 (4)	
O4	0.3323 (3)	0.40578 (13)	0.68055 (10)	0.0619 (6)	
O5	0.2499 (2)	0.48750 (10)	0.51066 (10)	0.0451 (5)	
O6	0.26769 (19)	0.34658 (10)	0.50243 (9)	0.0426 (4)	
O7	0.4062 (2)	0.22164 (10)	0.38831 (8)	0.0407 (4)	
O8	0.2868 (3)	0.34153 (14)	0.26162 (10)	0.0677 (6)	
N1	0.5100 (3)	0.61054 (12)	0.47244 (11)	0.0405 (5)	
C1	0.8052 (3)	0.53466 (15)	0.42806 (12)	0.0356 (6)	
H1	0.8947	0.4974	0.4248	0.043*	
C2	0.8586 (3)	0.61099 (16)	0.46832 (13)	0.0490 (7)	
H2B	0.9647	0.6238	0.4541	0.059*	
H2A	0.7929	0.6567	0.4543	0.059*	
C3	0.8527 (4)	0.60159 (17)	0.54915 (14)	0.0526 (7)	
H3B	0.8757	0.6539	0.5716	0.063*	
H3A	0.9318	0.5627	0.5643	0.063*	
C4	0.6923 (4)	0.57172 (16)	0.57444 (12)	0.0442 (7)	
C5	0.6682 (3)	0.48456 (14)	0.54468 (12)	0.0352 (6)	
H5	0.7477	0.4469	0.5632	0.042*	
C6	0.5037 (3)	0.45582 (14)	0.56507 (13)	0.0370 (6)	
H6	0.4612	0.4941	0.6007	0.044*	
C7	0.4063 (3)	0.46357 (14)	0.49505 (12)	0.0328 (5)	
C8	0.3806 (3)	0.38400 (14)	0.45413 (12)	0.0332 (6)	
C9	0.5323 (3)	0.33528 (13)	0.44938 (12)	0.0306 (5)	
H9	0.5529	0.3063	0.4947	0.037*	
C10	0.6707 (3)	0.39470 (14)	0.43015 (11)	0.0306 (5)	
C11	0.6670 (3)	0.48566 (13)	0.46105 (12)	0.0303 (5)	
C12	0.6726 (3)	0.39351 (15)	0.34574 (11)	0.0370 (6)	
H12B	0.6541	0.4481	0.3269	0.044*	
H12A	0.7729	0.3745	0.3283	0.044*	
C13	0.5428 (3)	0.33514 (15)	0.32151 (12)	0.0380 (6)	
H13	0.5727	0.3059	0.2774	0.046*	
C14	0.5350 (3)	0.27644 (14)	0.38592 (12)	0.0354 (6)	
H14	0.6318	0.2445	0.3877	0.042*	
C15	0.2966 (3)	0.39366 (16)	0.38029 (13)	0.0435 (6)	
H15A	0.2111	0.3547	0.3792	0.052*	
H15B	0.2509	0.4480	0.3790	0.052*	
C16	0.3906 (3)	0.38237 (17)	0.31056 (13)	0.0461 (7)	
H16	0.4155	0.4366	0.2909	0.055*	
C17	0.5051 (3)	0.52438 (13)	0.45053 (12)	0.0333 (6)	
H17	0.4752	0.5207	0.3996	0.040*	
C18	0.6936 (4)	0.57254 (18)	0.65786 (13)	0.0611 (8)	
H18B	0.7808	0.5412	0.6751	0.092*	
H18A	0.5985	0.5489	0.6756	0.092*	
H18C	0.7022	0.6280	0.6747	0.092*	
C19	0.5582 (4)	0.62569 (15)	0.54695 (13)	0.0483 (7)	
H19A	0.4687	0.6176	0.5781	0.058*	
H19B	0.5894	0.6826	0.5512	0.058*	
C20	0.3743 (4)	0.65879 (16)	0.45265 (16)	0.0589 (8)	
H20B	0.3870	0.7139	0.4715	0.071*	
H20A	0.2829	0.6351	0.4754	0.071*	
C21	0.3462 (5)	0.6638 (2)	0.37278 (19)	0.0874 (12)	
H21C	0.2639	0.7023	0.3633	0.131*	
H21A	0.3170	0.6108	0.3549	0.131*	
H21B	0.4399	0.6817	0.3491	0.131*	
C22	0.1671 (3)	0.41198 (16)	0.52121 (15)	0.0487 (7)	
H22B	0.1351	0.4069	0.5712	0.058*	
H22A	0.0744	0.4108	0.4912	0.058*	
C23	0.8951 (4)	0.5746 (2)	0.31168 (15)	0.0766 (11)	
H23C	0.9430	0.6253	0.3258	0.115*	
H23A	0.8619	0.5783	0.2623	0.115*	
H23B	0.9689	0.5308	0.3166	0.115*	
C24	0.4242 (3)	0.35893 (17)	0.65548 (13)	0.0404 (6)	
C25	0.4585 (4)	0.27508 (17)	0.68280 (14)	0.0532 (8)	
H25A	0.4412	0.2359	0.6450	0.080*	
H25B	0.3911	0.2630	0.7228	0.080*	
H25C	0.5653	0.2723	0.6983	0.080*	
C26	0.3097 (7)	0.3587 (3)	0.19060 (18)	0.134 (2)	
H26C	0.2695	0.4123	0.1800	0.201*	
H26A	0.2565	0.3188	0.1616	0.201*	
H26B	0.4194	0.3572	0.1801	0.201*	
C27	0.4061 (4)	0.16616 (17)	0.32879 (13)	0.0513 (7)	
H27B	0.3288	0.1245	0.3366	0.077*	
H27C	0.5071	0.1411	0.3245	0.077*	
H27A	0.3824	0.1955	0.2853	0.077*	

### Atomic displacement parameters (Å^2^)

**Table:**

	*U*^11^	*U*^22^	*U*^33^	*U*^12^	*U*^13^	*U*^23^
O1	0.0524 (11)	0.0455 (11)	0.0390 (9)	−0.0119 (10)	−0.0001 (8)	0.0130 (8)
O2	0.0335 (9)	0.0343 (10)	0.0493 (10)	0.0041 (8)	−0.0019 (8)	0.0066 (8)
O3	0.0483 (10)	0.0325 (9)	0.0332 (8)	−0.0016 (9)	0.0035 (8)	0.0076 (7)
O4	0.0654 (13)	0.0651 (14)	0.0551 (11)	0.0056 (12)	0.0227 (11)	0.0070 (11)
O5	0.0342 (10)	0.0422 (10)	0.0589 (11)	0.0059 (8)	0.0103 (9)	0.0064 (9)
O6	0.0327 (9)	0.0400 (9)	0.0551 (10)	−0.0040 (8)	0.0056 (8)	0.0105 (9)
O7	0.0462 (10)	0.0347 (9)	0.0412 (9)	−0.0115 (9)	−0.0030 (9)	−0.0020 (8)
O8	0.0757 (14)	0.0835 (16)	0.0440 (11)	−0.0151 (13)	−0.0221 (11)	0.0059 (11)
N1	0.0527 (13)	0.0256 (10)	0.0431 (11)	0.0030 (11)	0.0002 (10)	0.0030 (9)
C1	0.0363 (14)	0.0338 (13)	0.0368 (13)	−0.0052 (12)	−0.0024 (11)	0.0060 (11)
C2	0.0556 (18)	0.0410 (15)	0.0503 (15)	−0.0209 (15)	−0.0019 (14)	−0.0012 (13)
C3	0.067 (2)	0.0390 (15)	0.0517 (15)	−0.0172 (15)	−0.0127 (15)	−0.0005 (13)
C4	0.0646 (18)	0.0329 (14)	0.0352 (13)	−0.0091 (14)	−0.0014 (13)	−0.0020 (12)
C5	0.0421 (14)	0.0282 (12)	0.0355 (13)	−0.0027 (12)	−0.0029 (11)	0.0058 (11)
C6	0.0479 (16)	0.0260 (12)	0.0371 (13)	0.0016 (12)	0.0053 (13)	0.0024 (11)
C7	0.0304 (13)	0.0318 (12)	0.0361 (12)	0.0058 (11)	0.0034 (11)	0.0063 (11)
C8	0.0300 (13)	0.0312 (13)	0.0385 (12)	−0.0033 (11)	−0.0004 (11)	0.0072 (11)
C9	0.0339 (13)	0.0261 (12)	0.0319 (12)	−0.0020 (11)	−0.0004 (10)	0.0041 (10)
C10	0.0277 (12)	0.0282 (12)	0.0359 (12)	0.0011 (11)	−0.0002 (10)	0.0015 (11)
C11	0.0321 (13)	0.0259 (12)	0.0328 (12)	−0.0033 (11)	0.0007 (10)	0.0016 (10)
C12	0.0414 (14)	0.0329 (13)	0.0367 (12)	−0.0032 (13)	0.0038 (12)	−0.0003 (11)
C13	0.0470 (15)	0.0342 (13)	0.0327 (13)	−0.0055 (13)	−0.0005 (11)	−0.0003 (11)
C14	0.0378 (14)	0.0283 (11)	0.0400 (13)	−0.0033 (11)	−0.0051 (12)	0.0010 (12)
C15	0.0391 (14)	0.0396 (14)	0.0518 (14)	−0.0013 (13)	−0.0117 (13)	0.0054 (13)
C16	0.0513 (17)	0.0431 (15)	0.0440 (14)	−0.0040 (15)	−0.0096 (13)	0.0111 (13)
C17	0.0395 (14)	0.0279 (12)	0.0326 (12)	0.0005 (12)	0.0014 (11)	0.0032 (10)
C18	0.090 (2)	0.0506 (17)	0.0425 (14)	−0.0108 (19)	−0.0051 (16)	−0.0062 (14)
C19	0.0699 (19)	0.0313 (14)	0.0437 (14)	−0.0010 (14)	0.0055 (14)	−0.0057 (13)
C20	0.068 (2)	0.0358 (15)	0.072 (2)	0.0140 (15)	0.0056 (17)	0.0078 (15)
C21	0.113 (3)	0.063 (2)	0.086 (2)	0.029 (2)	−0.034 (2)	0.002 (2)
C22	0.0339 (14)	0.0512 (16)	0.0609 (16)	−0.0010 (14)	0.0069 (13)	0.0087 (15)
C23	0.079 (2)	0.099 (3)	0.0519 (16)	−0.037 (2)	0.0130 (18)	0.0120 (19)
C24	0.0430 (16)	0.0467 (16)	0.0317 (13)	−0.0132 (14)	−0.0037 (12)	0.0030 (13)
C25	0.067 (2)	0.0476 (16)	0.0452 (15)	−0.0197 (16)	−0.0031 (14)	0.0107 (14)
C26	0.155 (4)	0.180 (5)	0.067 (2)	−0.036 (4)	−0.052 (3)	0.031 (3)
C27	0.065 (2)	0.0427 (15)	0.0461 (15)	−0.0136 (16)	−0.0111 (15)	−0.0010 (13)

### Geometric parameters (Å, °)

**Table:**

O1—C1	1.437 (3)	C10—C11	1.591 (3)
O1—C23	1.405 (3)	C10—C12	1.565 (3)
O2—H2	0.8200	C11—C17	1.537 (3)
O2—C10	1.436 (3)	C12—H12B	0.9700
O3—C6	1.453 (3)	C12—H12A	0.9700
O3—C24	1.341 (3)	C12—C13	1.531 (3)
O4—C24	1.192 (3)	C13—H13	0.9800
O5—C7	1.426 (3)	C13—C14	1.532 (3)
O5—C22	1.435 (3)	C13—C16	1.529 (4)
O6—C8	1.453 (3)	C14—H14	0.9800
O6—C22	1.415 (3)	C15—H15A	0.9700
O7—C14	1.421 (3)	C15—H15B	0.9700
O7—C27	1.427 (3)	C15—C16	1.534 (4)
O8—C16	1.435 (3)	C16—H16	0.9800
O8—C26	1.360 (4)	C17—H17	0.9800
N1—C17	1.464 (3)	C18—H18B	0.9600
N1—C19	1.463 (3)	C18—H18A	0.9600
N1—C20	1.452 (3)	C18—H18C	0.9600
C1—H1	0.9800	C19—H19A	0.9700
C1—C2	1.522 (3)	C19—H19B	0.9700
C1—C11	1.555 (3)	C20—H20B	0.9700
C2—H2B	0.9700	C20—H20A	0.9700
C2—H2A	0.9700	C20—C21	1.502 (4)
C2—C3	1.507 (3)	C21—H21C	0.9600
C3—H3B	0.9700	C21—H21A	0.9600
C3—H3A	0.9700	C21—H21B	0.9600
C3—C4	1.532 (4)	C22—H22B	0.9700
C4—C5	1.539 (3)	C22—H22A	0.9700
C4—C18	1.546 (3)	C23—H23C	0.9600
C4—C19	1.534 (4)	C23—H23A	0.9600
C5—H5	0.9800	C23—H23B	0.9600
C5—C6	1.533 (4)	C24—C25	1.488 (4)
C5—C11	1.550 (3)	C25—H25A	0.9600
C6—H6	0.9800	C25—H25B	0.9600
C6—C7	1.548 (3)	C25—H25C	0.9600
C7—C8	1.520 (3)	C26—H26C	0.9600
C7—C17	1.543 (3)	C26—H26A	0.9600
C8—C9	1.527 (3)	C26—H26B	0.9600
C8—C15	1.554 (3)	C27—H27B	0.9600
C9—H9	0.9800	C27—H27C	0.9600
C9—C10	1.573 (3)	C27—H27A	0.9600
C9—C14	1.518 (3)		
			
O1—C1—H1	107.6	C8—C7—C6	115.28 (18)
O1—C1—C2	107.41 (18)	C8—C7—C17	111.23 (18)
O1—C1—C11	109.12 (19)	C8—C9—H9	110.8
O1—C23—H23C	109.5	C8—C9—C10	109.54 (18)
O1—C23—H23A	109.5	C8—C15—H15A	107.5
O1—C23—H23B	109.5	C8—C15—H15B	107.5
O2—C10—C9	108.35 (17)	C9—C8—C15	113.3 (2)
O2—C10—C11	108.27 (18)	C9—C10—C11	118.58 (19)
O2—C10—C12	105.95 (19)	C9—C14—C13	102.08 (18)
O3—C6—C5	108.5 (2)	C9—C14—H14	108.8
O3—C6—H6	108.2	C10—O2—H2	109.5
O3—C6—C7	117.9 (2)	C10—C9—H9	110.8
O3—C24—C25	109.8 (2)	C10—C12—H12B	110.3
O4—C24—O3	124.2 (2)	C10—C12—H12A	110.3
O4—C24—C25	126.0 (2)	C11—C1—H1	107.6
O5—C7—C6	111.04 (19)	C11—C5—H5	111.2
O5—C7—C8	101.45 (19)	C11—C17—C7	99.38 (17)
O5—C7—C17	116.62 (18)	C11—C17—H17	109.5
O5—C22—H22B	110.1	C12—C10—C9	103.11 (19)
O5—C22—H22A	110.1	C12—C10—C11	111.82 (18)
O6—C8—C7	98.53 (17)	C12—C13—H13	110.9
O6—C8—C9	112.58 (18)	H12B—C12—H12A	108.6
O6—C8—C15	106.09 (18)	C13—C12—C10	107.1 (2)
O6—C22—O5	108.19 (19)	C13—C12—H12B	110.3
O6—C22—H22B	110.1	C13—C12—H12A	110.3
O6—C22—H22A	110.1	C13—C14—H14	108.8
O7—C14—C9	111.21 (19)	C13—C16—C15	113.4 (2)
O7—C14—C13	116.8 (2)	C13—C16—H16	108.6
O7—C14—H14	108.8	C14—O7—C27	112.04 (19)
O7—C27—H27B	109.5	C14—C9—C8	112.75 (19)
O7—C27—H27C	109.5	C14—C9—H9	110.8
O7—C27—H27A	109.5	C14—C9—C10	101.69 (18)
O8—C16—C13	112.3 (2)	C14—C13—C12	101.07 (18)
O8—C16—C15	105.2 (2)	C14—C13—H13	110.9
O8—C16—H16	108.6	C15—C16—H16	108.6
O8—C26—H26C	109.5	H15A—C15—H15B	107.0
O8—C26—H26A	109.5	C16—C13—C12	110.2 (2)
O8—C26—H26B	109.5	C16—C13—H13	110.9
N1—C17—C7	119.00 (19)	C16—C13—C14	112.4 (2)
N1—C17—C11	109.5 (2)	C16—C15—C8	119.1 (2)
N1—C17—H17	109.5	C16—C15—H15A	107.5
N1—C19—C4	115.3 (2)	C16—C15—H15B	107.5
N1—C19—H19A	108.4	C17—C7—C6	101.83 (19)
N1—C19—H19B	108.4	C17—C11—C1	115.26 (18)
N1—C20—H20B	108.7	C17—C11—C5	97.91 (18)
N1—C20—H20A	108.7	C17—C11—C10	110.82 (19)
N1—C20—C21	114.0 (3)	H18B—C18—H18A	109.5
C1—C2—H2B	108.9	H18B—C18—H18C	109.5
C1—C2—H2A	108.9	H18A—C18—H18C	109.5
C1—C11—C10	108.84 (18)	C19—N1—C17	115.62 (19)
C2—C1—H1	107.6	C19—C4—C5	108.1 (2)
C2—C1—C11	117.2 (2)	C19—C4—C18	109.4 (2)
C2—C3—H3B	109.3	H19A—C19—H19B	107.5
C2—C3—H3A	109.3	C20—N1—C17	115.3 (2)
C2—C3—C4	111.5 (2)	C20—N1—C19	111.9 (2)
H2B—C2—H2A	107.7	C20—C21—H21C	109.5
C3—C2—C1	113.2 (2)	C20—C21—H21A	109.5
C3—C2—H2B	108.9	C20—C21—H21B	109.5
C3—C2—H2A	108.9	H20B—C20—H20A	107.6
C3—C4—C5	107.7 (2)	C21—C20—H20B	108.7
C3—C4—C18	107.3 (2)	C21—C20—H20A	108.7
C3—C4—C19	112.8 (2)	H21C—C21—H21A	109.5
H3B—C3—H3A	108.0	H21C—C21—H21B	109.5
C4—C3—H3B	109.3	H21A—C21—H21B	109.5
C4—C3—H3A	109.3	C22—O6—C8	103.91 (17)
C4—C5—H5	111.2	H22B—C22—H22A	108.4
C4—C5—C11	110.40 (19)	C23—O1—C1	113.8 (2)
C4—C18—H18B	109.5	H23C—C23—H23A	109.5
C4—C18—H18A	109.5	H23C—C23—H23B	109.5
C4—C18—H18C	109.5	H23A—C23—H23B	109.5
C4—C19—H19A	108.4	C24—O3—C6	118.5 (2)
C4—C19—H19B	108.4	C24—C25—H25A	109.5
C5—C4—C18	111.6 (2)	C24—C25—H25B	109.5
C5—C6—H6	108.2	C24—C25—H25C	109.5
C5—C6—C7	105.31 (19)	H25A—C25—H25B	109.5
C5—C11—C1	113.22 (19)	H25A—C25—H25C	109.5
C5—C11—C10	110.43 (18)	H25B—C25—H25C	109.5
C6—C5—C4	108.5 (2)	C26—O8—C16	115.2 (3)
C6—C5—H5	111.2	H26C—C26—H26A	109.5
C6—C5—C11	104.1 (2)	H26C—C26—H26B	109.5
C7—O5—C22	104.93 (18)	H26A—C26—H26B	109.5
C7—C6—H6	108.2	H27B—C27—H27C	109.5
C7—C8—C9	110.47 (19)	H27B—C27—H27A	109.5
C7—C8—C15	114.85 (19)	H27C—C27—H27A	109.5
C7—C17—H17	109.5		
			
O1—C1—C2—C3	160.6 (2)	C8—C15—C16—O8	142.5 (2)
O1—C1—C11—C5	−158.10 (19)	C8—C15—C16—C13	19.4 (3)
O1—C1—C11—C10	78.7 (2)	C9—C8—C15—C16	−20.5 (3)
O1—C1—C11—C17	−46.5 (3)	C9—C10—C11—C1	−173.89 (18)
O2—C10—C11—C1	62.2 (2)	C9—C10—C11—C5	61.2 (3)
O2—C10—C11—C5	−62.6 (2)	C9—C10—C11—C17	−46.1 (3)
O2—C10—C11—C17	−170.02 (17)	C9—C10—C12—C13	−0.8 (2)
O2—C10—C12—C13	112.9 (2)	C10—C9—C14—O7	−173.61 (18)
O3—C6—C7—O5	94.0 (2)	C10—C9—C14—C13	−48.3 (2)
O3—C6—C7—C8	−20.7 (3)	C10—C11—C17—N1	−174.08 (17)
O3—C6—C7—C17	−141.2 (2)	C10—C11—C17—C7	60.5 (2)
O5—C7—C8—O6	−46.64 (19)	C10—C12—C13—C14	−28.0 (2)
O5—C7—C8—C9	−164.70 (18)	C10—C12—C13—C16	91.0 (2)
O5—C7—C8—C15	65.6 (2)	C11—C1—C2—C3	37.4 (3)
O5—C7—C17—N1	49.4 (3)	C11—C5—C6—O3	113.0 (2)
O5—C7—C17—C11	167.90 (19)	C11—C5—C6—C7	−14.1 (2)
O6—C8—C9—C10	−152.60 (18)	C11—C10—C12—C13	−129.3 (2)
O6—C8—C9—C14	94.9 (2)	C12—C10—C11—C1	−54.1 (3)
O6—C8—C15—C16	−144.5 (2)	C12—C10—C11—C5	−178.99 (19)
C1—C2—C3—C4	−52.2 (3)	C12—C10—C11—C17	73.6 (2)
C1—C11—C17—N1	−49.9 (2)	C12—C13—C14—O7	168.94 (19)
C1—C11—C17—C7	−175.36 (19)	C12—C13—C14—C9	47.4 (2)
C2—C1—C11—C5	−35.8 (3)	C12—C13—C16—O8	156.1 (2)
C2—C1—C11—C10	−159.0 (2)	C12—C13—C16—C15	−84.8 (2)
C2—C1—C11—C17	75.8 (3)	C14—C9—C10—O2	−82.3 (2)
C2—C3—C4—C5	65.4 (3)	C14—C9—C10—C11	153.85 (18)
C2—C3—C4—C18	−174.4 (2)	C14—C9—C10—C12	29.7 (2)
C2—C3—C4—C19	−53.9 (3)	C14—C13—C16—O8	−92.1 (2)
C3—C4—C5—C6	−176.2 (2)	C14—C13—C16—C15	27.0 (3)
C3—C4—C5—C11	−62.7 (3)	C15—C8—C9—C10	87.0 (2)
C3—C4—C19—N1	79.8 (3)	C15—C8—C9—C14	−25.5 (3)
C4—C5—C6—O3	−129.4 (2)	C16—C13—C14—O7	51.5 (3)
C4—C5—C6—C7	103.5 (2)	C16—C13—C14—C9	−70.0 (2)
C4—C5—C11—C1	48.2 (3)	C17—N1—C19—C4	40.2 (3)
C4—C5—C11—C10	170.6 (2)	C17—N1—C20—C21	−61.1 (3)
C4—C5—C11—C17	−73.7 (2)	C17—C7—C8—O6	−171.33 (17)
C5—C4—C19—N1	−39.2 (3)	C17—C7—C8—C9	70.6 (2)
C5—C6—C7—O5	−144.84 (19)	C17—C7—C8—C15	−59.1 (3)
C5—C6—C7—C8	100.5 (2)	C18—C4—C5—C6	66.3 (3)
C5—C6—C7—C17	−20.0 (2)	C18—C4—C5—C11	179.9 (2)
C5—C11—C17—N1	70.5 (2)	C18—C4—C19—N1	−160.9 (2)
C5—C11—C17—C7	−55.0 (2)	C19—N1—C17—C7	55.1 (3)
C6—O3—C24—O4	0.7 (4)	C19—N1—C17—C11	−58.1 (3)
C6—O3—C24—C25	−178.0 (2)	C19—N1—C20—C21	164.0 (3)
C6—C5—C11—C1	164.53 (18)	C19—C4—C5—C6	−54.0 (2)
C6—C5—C11—C10	−73.1 (2)	C19—C4—C5—C11	59.5 (3)
C6—C5—C11—C17	42.6 (2)	C20—N1—C17—C7	−78.1 (3)
C6—C7—C8—O6	73.4 (2)	C20—N1—C17—C11	168.7 (2)
C6—C7—C8—C9	−44.6 (3)	C20—N1—C19—C4	174.9 (2)
C6—C7—C8—C15	−174.4 (2)	C22—O5—C7—C6	−89.4 (2)
C6—C7—C17—N1	−71.7 (3)	C22—O5—C7—C8	33.7 (2)
C6—C7—C17—C11	46.9 (2)	C22—O5—C7—C17	154.6 (2)
C7—O5—C22—O6	−7.8 (3)	C22—O6—C8—C7	42.0 (2)
C7—C8—C9—C10	−43.5 (2)	C22—O6—C8—C9	158.4 (2)
C7—C8—C9—C14	−155.99 (18)	C22—O6—C8—C15	−77.1 (2)
C7—C8—C15—C16	107.8 (3)	C23—O1—C1—C2	75.5 (3)
C8—O6—C22—O5	−22.9 (2)	C23—O1—C1—C11	−156.5 (2)
C8—C7—C17—N1	165.0 (2)	C24—O3—C6—C5	140.1 (2)
C8—C7—C17—C11	−76.4 (2)	C24—O3—C6—C7	−100.3 (2)
C8—C9—C10—O2	158.19 (18)	C26—O8—C16—C13	−86.4 (4)
C8—C9—C10—C11	34.3 (3)	C26—O8—C16—C15	149.8 (3)
C8—C9—C10—C12	−89.8 (2)	C27—O7—C14—C9	179.6 (2)
C8—C9—C14—O7	−56.4 (2)	C27—O7—C14—C13	63.0 (3)
C8—C9—C14—C13	68.9 (2)		

### Hydrogen-bond geometry (Å, °)

**Table:**

*D*—H···*A*	*D*—H	H···*A*	*D*···*A*	*D*—H···*A*
O2—H2···O7^i^	0.82	2.58	3.311 (2)	150

Symmetry codes: (i) *x*+1/2, −*y*+1/2, −*z*+1.
